# O.4.1-1 Impact of a pre-pandemic, tele-exercise, fall-prevention intervention on outcomes in older adults

**DOI:** 10.1093/eurpub/ckad133.166

**Published:** 2023-09-11

**Authors:** Dina Lynn Jones, Maura Robinson, McKinzey Dierkes, Terry Kit Selfe

**Affiliations:** West Virginia University, USA; West Virginia University, USA; West Virginia University, USA; University of Florida, USA; dljones@hsc.wvu.edu

## Abstract

**Purpose:**

This pre-pandemic study used an alternative delivery mode to provide an evidence-based, fall-prevention exercise intervention to older adults. We delivered Tai Ji Quan: Moving for Better Balance^®^ (TJQMBB) via tele-exercise and determined its impact on self-reported and performance-based outcomes in older adults.

**Methods:**

Five community sites (3 urban, 2 rural) held 6, 1-hour, synchronous TJQMBB classes, over the internet, twice weekly for 16 weeks (32 hours). Baseline and 16-week self-reported data included fear of falling (Falls Efficacy Scale), depression (Geriatric Depression Scale-Short), physical and mental health-related quality of life (HRQOL) (SF-12 PCS and MCS), and total minutes of physical activity per week. Performance-based outcomes included a 5-Meter Walk Test (gait speed), the Timed Up-and-Go Test (mobility), the 4-Square Step Test (balance), and the 5-Times Sit-to-Stand Test (leg strength). Height and weight were used to calculate body mass index (BMI). Paired t-tests and Friedman 2-Way ANOVA tests examined changes in outcomes between baseline and 16 weeks.

**Results:**

Sixty-three (79.7%) of 79 people were eligible, of which 52 (82.5%) enrolled. Participants were a mean ± SD age of 68.5 ± 7.7 years old, primarily white (96.2%), and female (80.8%). There were no significant improvements in fear of falling (X^2^_r_ =3.8, p = 0.15), depression (X^2^_r_ =0.19, p = 0.91), physical (X^2^_r_ =1.2, p = 0.56) or mental (X^2^_r_ =0.31, p = 0.86) HRQOL, or physical activity (X^2^_r_ =3.7, p = 0.16). Gait speed (meters/second) significantly improved from baseline (1.1 ± 0.2) to 16 weeks (1.2 ± 0.2) (p = 0.02). Balance, leg strength, and BMI also improved significantly (balance [seconds]: baseline 10.9 ± 2.4, 16 weeks 10.2 ± 2.5, p = 0.045; strength [seconds]: baseline 14.7 ± 5.7, 16 weeks 12.4 ± 2.9, p = 0.01; BMI [kg/m^2^]: baseline 30.8 ± 6.3, 16 weeks 30.2 ± 5.6, p < 0.01). There were no changes in mobility (p = 0.16).

**Conclusions:**

This tele-TJQMBB intervention improved physical function (gait speed, balance, leg strength) and BMI in older adults. Tele-TJQMBB provided an alternative delivery mode that could help overcome the barrier of identifying experienced instructors in rural areas, and could expand TJQMBB’s reach into the older adult population. Future comparative effectiveness studies could establish if outcomes are equivalent between in-person and tele-exercise delivery modes.

